# Efficacy of Semen Microencapsulation Technique on the Quality of Spermatozoa From Canindé Goats Kept Under Refrigeration for Three Days

**DOI:** 10.1111/rda.70141

**Published:** 2025-11-06

**Authors:** Francisca Kaline Pereira de Souza, Maiana Silva Chaves, Camila Helen Mendonça Rodrigues, Irving Mitchell Laines Arcce, Satish Kumar, Luciana Magalhães Melo, Vicente José de Figueirêdo Freitas

**Affiliations:** ^1^ Laboratory of Physiology and Control of Reproduction, Faculty of Veterinary State University of Ceará Fortaleza Brazil; ^2^ Center for Science, Education and Technology State University of Ceará Tauá Brazil

**Keywords:** caprine, CASA, conservation, encapsulation, sperm

## Abstract

For approximately 40 years, microencapsulation technology has been utilised across various species due to its ability to release semen gradually after artificial insemination. This study aimed to establish the use of the alginate microencapsulation procedure for goat semen and to investigate whether this method enhances longevity during cold storage compared to the traditional straw method. Semen was collected from Canindé bucks and analysed using Computer‐Assisted Semen Analysis (CASA). The semen was then diluted in a commercial extender and packaged either in straws or microcapsules (using 1% sodium alginate). Both groups were refrigerated at 4°C–5°C and assessed at 24, 48 and 72 h after dilution. The evaluation included assessments of sperm viability, abnormalities, membrane integrity, and DNA integrity. Data were analysed using repeated‐measures ANOVA at *p* < 0.05. Concerning the parameters straight line (VSL) and average path (VAP), no statistical differences (*p* > 0.05) were observed. However, the microcapsule group showed significantly higher results (*p* < 0.05) for straightness (STR), beat cross frequency (BCF), and wobble (WOB) at 24 and 48 h of storage. Sperm viability was also higher (*p* < 0.05) in the microcapsule group at 24 and 48 h of storage. In conclusion, this study demonstrates the feasibility of microencapsulating goat semen. Further in vivo and/or in vitro fertility trials are needed to confirm these findings.

## 1 Introduction

Northeast Brazil has approximately 13 million goats, accounting for 91% of the country's total goat population (Brazilian Institute of Geography and Statistics 2023). Goat herds in the region are diverse, including high milk‐producing breeds such as the Saanen and Alpine, as well as breeds naturally adapted to the local environment. Among these, Canindé goats play an important role in subsistence farming. Unfortunately, uncontrolled crossbreeding with exotic breeds has led to genetic degeneration, placing the Canindé goats at risk and classifying them as endangered (Mariante and Egito 2002).

A variety of reproductive biotechnology techniques have been utilised to conserve domestic animal breeds, including artificial insemination (AI) and multiple ovulation and embryo transfer (MOET) (Mara et al. 2013). Recent advancements have led to the development of more effective and practical methods, making AI and MOET viable options for goats (Souza‐Fabjan et al. 2023). One of the benefits of AI is that it minimises the risk of sexually transmitted diseases and allows for the preservation of reproductive potential through semen cryopreservation (Leboeuf et al. 2000). During AI, millions of sperm are deposited into the female reproductive system. However, only a small fraction reaches the fertilisation site, which lowers the overall efficiency of the technique. Research has observed that maintaining an adequate number of sperm during ovulation can significantly enhance fertilisation success rates (Fraser 1998).

When AI is performed more than 24 h after semen collection, the use of refrigerated semen is recommended (Mocé et al. 2020). However, this approach has its drawbacks, as prolonged storage reduces sperm viability, compromising the success rates of AI, particularly regarding sperm longevity (Leboeuf et al. 2000). To overcome this problem, sperm microencapsulation has emerged as a promising solution. This technique involves coating sperm cells with a semipermeable polymeric membrane that allows for the controlled release of viable sperm, potentially extending their availability in the female reproductive tract (Nebel et al. 1985; Vigo et al. 2009).

The controlled release of encapsulated spermatozoa into the uterus during AI may decrease retrograde movement and prevent premature capacitation. Thus, it could increase the time available for oocyte fertilisation (Nebel et al. 1985). The material used to form these capsules can be a single type or a combination of materials, such as alginate, cellulose sulphate and liposomes (Weber et al. 2006). Alginate microencapsulation is frequently used to maintain bioactive components because its structure allows the exchange of nutrients and metabolites (Uludag et al. 2000). Alginate microencapsulation has the potential to protect spermatozoa from contamination risks associated with foreign substances, whether they are other spermatozoa or genetic material (Herrler et al. 2006). Moreover, microcapsules protect the spermatozoa against environmental stressors such as temperature changes, mechanical impacts, and the accumulation of damaging compounds (Dubey 2009). Alginates are derived from brown algae and consist of unbranched polysaccharides made up of guluronic (G) and mannuronic (M) acid residues. These can form either homopolymers or heteropolymers. Gelation occurs when divalent ions like Ca^2+^ or Ba^2+^ interact with G‐blocks and create a three‐dimensional structure. The mechanical properties and macrostructure of the alginate gel depend on the G/M ratio and the type of ions used. Notably, barium ions combined with a high proportion of G‐blocks produce stronger gels (Faustini 2011). Therefore, this study aimed to establish the use of the alginate microencapsulation procedure for goat semen and to investigate whether this method enhances longevity during cold storage compared to the traditional straw method.

## 2 Material and Methods

### 2.1 Ethical Approval

The Ethics Committee on the Use of Animals (# 31032.004688/2024‐39) approved this experiment, which follows the guidelines for experimental handling established by Sandøe et al. (2008).

### 2.2 Local and Animals

This study was conducted at the State University of Ceará facilities located in Fortaleza, Brazil (3°43′58″ S, 38°31′37″ W). Sexually mature Canindé bucks (*n* = 2, age of 2–4 years, and weight of 35–45 kg) were used as semen donors. Goats were healthy, housed in individual barns and fed concentrate (minimum 18% CP). Minerals and freshwater were available ad libitum.

### 2.3 Semen Collection and Initial Evaluation

**FIGURE 1:**
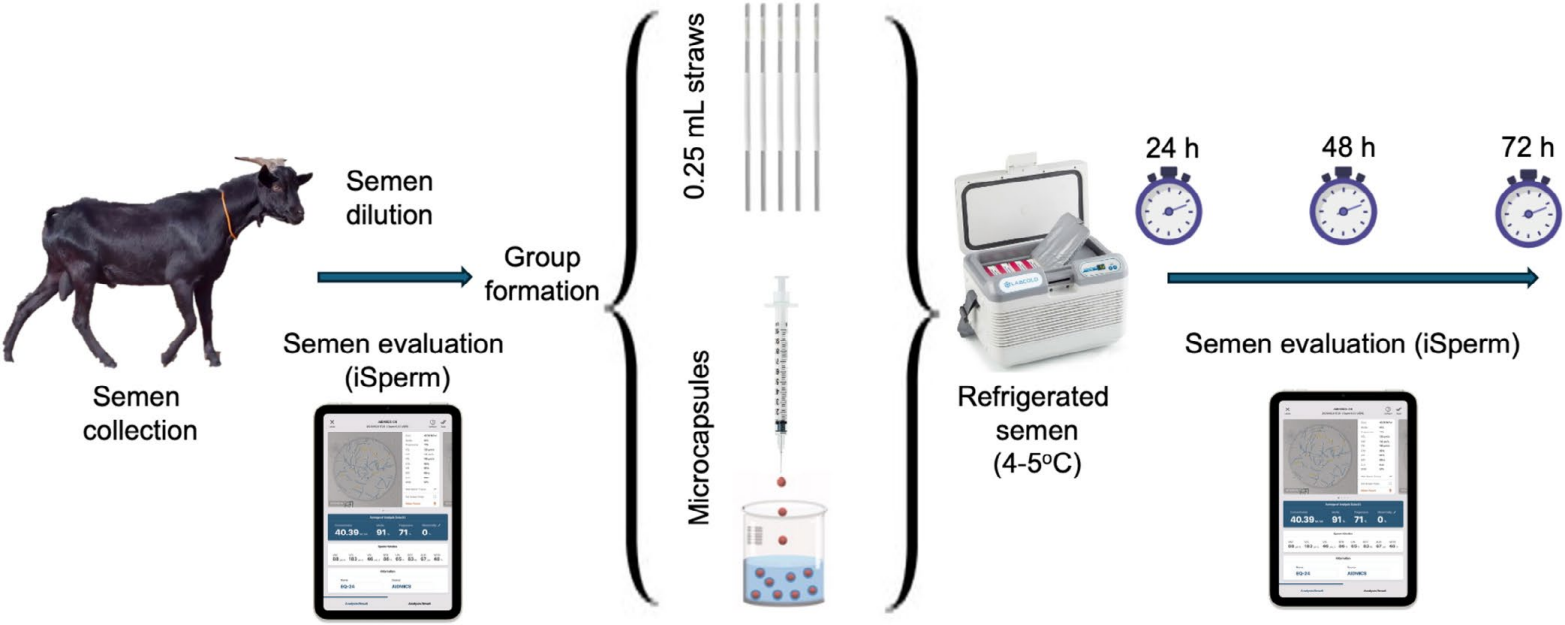
Experimental design detailing the steps from collection to evaluation of semen packaged in straws or microcapsules.

Semen was collected twice a week by the artificial vagina method. Sperms were analysed using CASA equipment (iSperm, Aidmics Biotechnology, Taipei City, Taiwan), which has software developed exclusively for goats and as per industrial conventions. Ejaculates (*n* = 22) with more than 70% progressive motility were selected for use. The semen was then diluted with a commercial extender (Optidux, Reprodux, Campinas, Brazil) to achieve a concentration of 100 × 10^6^ spermatozoa/mL. After dilution, the semen was reevaluated using the iSperm system (0 h) and subsequently packaged in straws or microcapsules in 2 mL Eppendorf. The two groups were refrigerated at 4°C–5°C and assessed at 24, 48 and 72 h after thawing (Figure 1).

### 2.4 Microcapsules Preparation

For this step, the protocol described by Hernández Pichardo et al. (2021) was used with some modifications. Briefly, the pre‐diluted semen with Optidux extender (100 million spermatozoa/mL) was mixed with a 1% sodium alginate solution (prepared in autoclaved distilled water) in a 1:1 ratio. Then, using a 1 mL syringe with a 26G needle, the mixture was dripped from a height of 15 cm (tip of the needle to the surface of the solution) into a 50 mM barium chloride solution (diluted in autoclaved distilled water). The microcapsules were allowed to remain in this solution for 30 s to undergo polymerisation. After this period, the microcapsules were washed twice with saline solution (0.9% NaCl). Subsequently, 20 microcapsules were placed in a 2 mL Eppendorf tube containing 500 μL Optidux extender. For a detailed structural verification, the microcapsules were observed under light‐sheet microscopy (Carl Zeiss Microscopy Deutschland GmbH, Oberkochen, Germany) following the manufacturer's instructions.

### 2.5 | Thawing of Semen Straw and Microcapsules

The semen straw thawed at 37°C for 30 s. For microcapsules, a 2 mL Eppendorf tube containing 20 microcapsules with extender was placed at 37.5°C for 30 min (Hernández Pichardo et al. 2021). The microcapsule structure was then disintegrated by gentle pipetting (Pruß et al. 2022).

### 2.6 Computer‐Assisted Sperm Analysis

The semen was evaluated 24, 48 and 72 h after thawing. For each evaluation, 7.5 μL of semen was placed into an iSperm device, and four randomly selected fields were analysed. According to the study by Cox et al. (2006), the following sperm parameters were recorded: progressive motility (PM, %), curved line velocity (VCL, μm/s), straight‐line velocity (VSL, μm/s), average path velocity (VAP, μm/s), amplitude of lateral head displacement (ALH, μm), linearity (LIN), straightness (STR), beat cross frequency (BCF, Hz) and wobble (WOB, %).

### 2.7 Sperm Viability, Abnormality, Membrane Integrity and DNA Integrity

For each refrigeration period (24, 48 and 72 h), semen samples were analysed. Sperm viability was determined in semen samples (10 μL) stained with a dual stain of eosin‐nigrosine (5 μL) and smeared on a glass slide (Appell and Evans 1977). About 100 spermatozoa were examined using a light microscope (Nikon, Eclipse E400, Japan) at 400× magnification. The sperm that showed colour were considered dead, while those that showed no intensity of colour were considered alive. On the same slide, the percentage of total sperm abnormalities was calculated (Chandler et al. 1988). Additionally, sperm plasma membrane integrity was assessed by the hypo‐osmotic swelling test. Briefly, 10 μL of semen was mixed with hypo‐osmotic solution (HOS) containing sodium citrate and fructose in distilled water and incubated at 37°C for 30 min on the slide. Two hundred sperm cells were counted under a microscope at 400× magnification to observe cells with swelled or curled tails (Neild et al. 1999). Spermatozoa DNA integrity percentage was determined by the sperm chromatin dispersion test, as described by Fernández et al. (2003). Briefly, agarose was boiled for 5 min, then 50 μL was kept at 37°C in a water bath for 5 min with semen (25 μL). An aliquot (2 μL) of solution was placed under a coverslip on a pre‐prepared slide. The slides were then placed in a refrigerator at 4°C for 5 min. After removing the coverslip, the slides were treated with hydrochloric acid (HCl) for 7 min. They were subsequently treated with a lysis solution for 25 min, followed by immersion in distilled water for 5 min. Finally, the slides were placed in 70%, 90% and 95% alcohol solutions for 2 min each, respectively.

The slides were then dried at room temperature and stained with the Panoptic kit (Renylab, Barbacena, Brazil). The slides were rewashed in distilled water and dried at room temperature. The DNA integrity percentage of spermatozoa was determined by a large halo of chromatin dispersion around the sperm head, using 200 spermatozoa per slide.

### 2.8 Statistical Analysis

The Shapiro–Wilk test was conducted to assess the normality of the data distribution. After confirming the normality of the data, they were analysed using repeated‐measures ANOVA, with treatment type (straws vs. microcapsules) as a fixed factor and semen refrigeration time (24, 48 and 72 h) as a repeated‐measures variable. Significant differences were determined at a 5% probability level using Tukey's Multiple Comparison Test. The data were graphed using JMP version 18.0.1 (JMP Statistical Discovery LLC, Cary, NC, USA).

## 3 | Results

Before the dilution procedure, semen presented the following mean (± SEM) values: 0.60 ± 0.07 mL; 4.50 ± 0.11; 5.86 ± 0.20; 10^6^ sperm/mL and 76.83% ± 1.52% for volume, mass motility, sperm concentration and progressive motility, respectively. Additionally, no statistical differences (*p* > 0.05) were observed in semen parameters between the two males used in the study.

**FIGURE 2:**
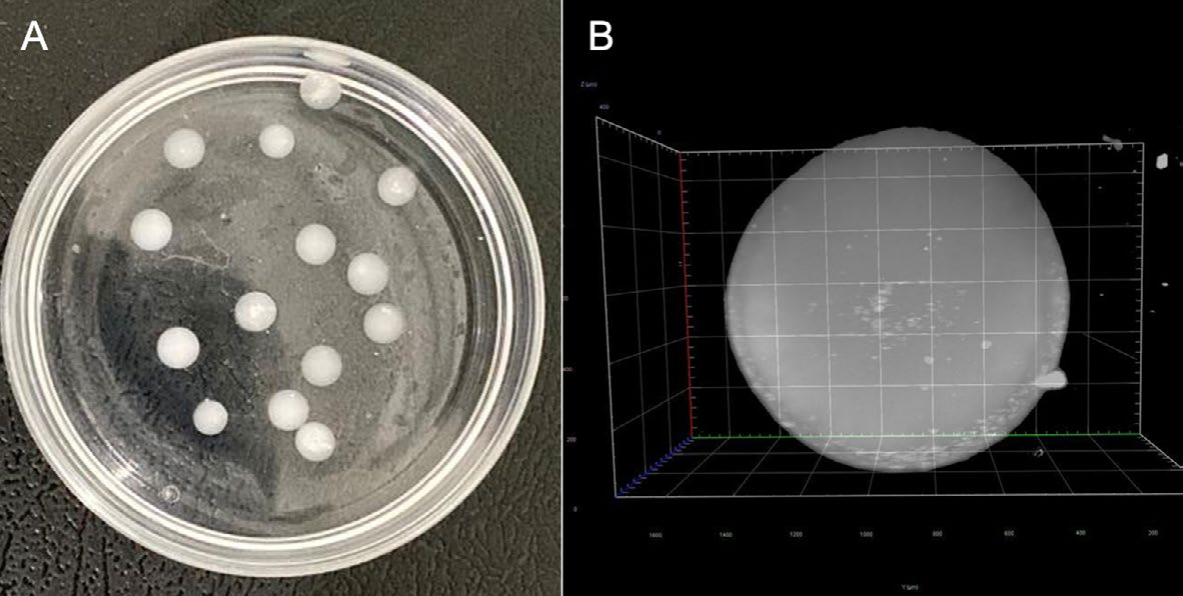
Microencapsulated goat semen: Newly formed microcapsules (A) and subsequently observed under light sheet microscopy (B).

Sixty microcapsules were produced for each ejaculate, with an approximate volume of 7.5 μL. The rigid spherical microcapsules are shown in Figure 2A, and the mean diameter (± SEM) of each capsule was 5224 ± 598 μm (Figure 2B). The uniformity of the microcapsules and the presence of sperm within them were confirmed (Figure 2B).

In general, the results obtained at the end of 72 h of refrigeration were significantly lower (*p* < 0.05) than those observed at the time of semen collection (Tables 1 and 2). Concerning the semen analysis by CASA, the results (mean ± SEM) are shown in Table 1. There were no statistical differences (*p* > 0.05) observed between the straw and microcapsule groups for VSL and VAP at any moments (24, 48 or 72 h).

**TABLE 1:** Sperm motion parameters of goat semen packaged in straws or microcapsules, stored refrigerated and evaluated by CASA at 24, 48 and 72 h of incubation.

Groups	0 h	24 h	48 h	72 h
Progressive motility (%)				
Fresh	73.1 ± 7.0^a^	—	—	—
Straw	—	75.5 ± 11.7^A,a^	59.7 ± 19.7^A,b^	34.0 ± 18.2^A,b^
Microcapsule	—	35.4 ± 10.2^B,b^	29.5 ± 11.9^B,b^	24.8 ± 12.6^A,b^
VCL (μm/s)				
Fresh	194.8 ± 26.4^a^	—	—	—
Straw	—	170.0 ± 15.8^A,a^	147.7 ± 27.0^A,b^	63.8 ± 12.2^A,b^
Microcapsule	—	151.4 ± 26.8^B,b^	128.4 ± 33.6^A,b^	57.1 ± 17.9^A,b^
VSL (μm/s)				
Fresh	110.9 ± 10.9^a^	—	—	—
Straw	—	61.2 ± 6.9^A,b^	49.4 ± 10.6^A,b^	49.0 ± 11.8^A,b^
Microcapsule	—	59.1 ± 9.7^A,b^	45.8 ± 13.9^A,b^	45.6 ± 12.8^A,b^
VAP (μm/s)				
Fresh	125.8 ± 11.7^a^	—	—	—
Straw	—	81.9 ± 6.2^A,b^	68.7 ± 10.8^A,b^	63.8 ± 12.2^A,b^
Microcapsule	—	78.2 ± 12.2^A,b^	63.4 ± 17.2^A,b^	57.1 ± 17.9^A,b^
ALH (μm)				
Fresh	10.7 ± 1.2^a^	—	—	—
Straw	—	12.9 ± 4.2^A,a^	11,4 ± 3,9^A,a^	9.5 ± 1.7^A,a^
Microcapsule		7.7 ± 1.8^A,a^	6.7 ± 1.6^B,b^	10.8 ± 11.1^A,a^
LIN (μm/s)				
Fresh	56.1 ± 7.4^a^	—	—	—
Straw	—	40.4 ± 11.2^A,a^	36.2 ± 13.4^A,b^	37.0 ± 7.7^A,b^
Microcapsule	—	42.2 ± 5.0^A,a^	39.6 ± 4.9^A,b^	47.0 ± 13.5^B,a^
STR (μm/s)				
Fresh	84.6 ± 4.9^a^	—	—	—
Straw	—	67.2 ± 13.0^A,b^	67.5 ± 10.2^A,b^	73.2 ± 9.2^A,a^
Microcapsule	—	76.3 ± 7.3^B,a^	76.3 ± 7.3^B,a^	73.3 ± 18.7^A,a^
BCF (Hz)				
Fresh	34.8 ± 2.3^a^	—	—	—
Straw	—	24.8 ± 4.6^A,b^	23.6 ± 4.4^A,b^	25.9 ± 1.9^A,b^
Microcapsule	—	33.0 ± 4.2^B,a^	32.8 ± 5.4^B,a^	28.1 ± 11.0^A,b^
WOB (%)				
Fresh	64.2 ± 6.1^a^	—	—	—
Straw	—	48.3 ± 3.1^A,b^	47.5 ± 4.5^A,b^	49.5 ± 4.3^A,b^
Microcapsule	—	53.5 ± 3.4^B,a^	50.5 ± 4.5^B,b^	50.2 ± 6.0^A,b^

*Note:* Values with different uppercase letters differ significantly (*p* < 0.05) between groups. Values with different lowercase letters differ significantly (*p* < 0.05) among incubation time.

**TABLE 2:** Viability, abnormalities, membrane integrity and DNA integrity of goat semen packaged in straws or microcapsules, stored refrigerated and evaluated at 24, 48 and 72 h of incubation.

Groups	0 h	24 h	48 h	72 h
Viability (%)				
Fresh	92.2 ± 2.9^a^	—	—	—
Straw	—	89.7 ± 3.3^A,b^	86.3 ± 3.7^A,b^	81.1 ± 6.8^A,b^
Microcapsule	—	91.7 ± 2.5^B,a^	89.9 ± 4.2^B,b^	84.0 ± 6.8^A,b^
Abnormality (%)				
Fresh	7.9 ± 4.3^a^	—	—	—
Straw	—	8.6 ± 4.1^A,a^	9.4 ± 4.1^A,b^	11.3 ± 4.7^A,b^
Microcapsule	—	7.6 ± 2.7^A,a^	9.0 ± 3.5^A,b^	10.7 ± 4.2^A,b^
Membrane integrity (%)				
Fresh	89.5 ± 2.1^a^	—	—	—
Straw	—	85.3 ± 3.6^A,b^	81.4 ± 4.8^A,b^	75.7 ± 7.8^A,b^
Microcapsule	—	87.4 ± 3.6^A,a^	83.3 ± 5.5^A,b^	78.7 ± 7.0^A,b^
DNA integrity				
Fresh	90.4 ± 4.0^a^	—	—	—
Straw	—	88.8 ± 4.2^A,b^	85.3 ± 5.0^A,b^	80.1 ± 7.1^A,b^
Microcapsule	—	80.5 ± 5.0^A,b^	85.3 ± 6.9^A,b^	80.4 ± 7.9^A,b^

*Note:* Values with different uppercase letters differ significantly (*p* < 0.05) between groups. Values with different lowercase letters differ significantly (*p* < 0.05) among incubation time.

On the other hand, the STR, BCF and WOB parameters for the microcapsule group were higher (*p* < 0.05) at 24 and 48 h. However, this difference was no longer identified (*p* > 0.05) at 72 h of refrigeration. Among the other CASA parameters, only the progressive motility was significantly higher (*p* < 0.05) in the straw group compared to the microcapsule group at both 24 and 48 h of refrigeration.

The results of sperm viability, abnormalities, membrane and DNA integrity are presented in Table 2. The viability of the microcapsule group was higher (*p* < 0.05) at 24 and 48 h compared to the straw group. All other variables showed no significant differences (*p* > 0.05) between the groups tested.

## 4 Discussion

In this study, we present a procedure for microencapsulation of goat semen using alginate with barium chloride as a gelation‐inducing agent. Sperm microencapsulation consists of coating sperm cells with a semipermeable polymeric membrane (Perteghella et al. 2015). The microcapsule structure can be composed of a single material or a combination of several, such as alginate, cellulose sulphate and liposomes (Olabisi 2015). Microcapsule formation depends directly on the concentration and properties of the alginate and gelling agent used. In our study, barium chloride was used, which forms more rigid and stable gels, promoting the production of spherical and homogeneous capsules (Nivethitha et al. 2024). This increased rigidity not only enhances the physical integrity of the microcapsules but also delays premature sperm capacitation, thus preserving their viability and functionality (Zhang and Gopalakrishnan 2005). Studies indicate that sperm encapsulated in barium alginate (BaAlg) maintain high motility and acrosomal integrity, in addition to presenting greater enzymatic activity over time (Vigo et al. 2002; Faustini et al. 2004).

Torre et al. (2002) observed that microencapsulated swine semen maintained at 38°C for 24 h did not negatively impact cellular metabolism or compromise acrosomal integrity. Additionally, the gradual release of gametes demonstrated the functional biodegradability of the capsules, which did not affect the quality of the semen (Torre et al. 2002). Alginate encapsulation is commonly used to maintain bioactive components because its structure facilitates the exchange of nutrients and metabolites (Rabanel et al. 2009). This technique has been successfully applied in several species, such as cattle (Nebel et al. 1985), swine (Faustini et al. 2012), buffalo (Perteghella et al. 2017) and horses (Pruß et al. 2022). To date, only one study on goats has standardised the protocol for semen microencapsulation (Nivethitha et al. 2024), but it did not test sperm kinetics in response to treatments. In our experiment on microcapsule formation, we used 1% alginate because concentrations below 1% result in non‐spherical capsules, likely due to a lack of sufficient carboxyl groups during the gelation process (Nivethitha et al. 2024).

Firstly, the results obtained immediately after semen collection align with the expected parameters for goats (Liu et al. 2016; Anand and Yadav 2016), including those verified for the Canindé breed (Câmara et al. 2019). To the best of our knowledge, there are no studies that have evaluated microcapsules through light sheet microscopy. However, using this technique, it was possible to verify in detail the structure and homogeneity of the microcapsules. Over the years, light sheet microscopy has undergone significant advancements, transforming its capabilities and broadening its applications. This approach allows for the creation of high‐resolution 3D reconstructions of structures, such as microcapsules, enabling the observation of increased spatial accuracy (Raju et al. 2025).

In this study, we demonstrated that after cryopreservation, sperm from both groups (straw and microcapsule) showed high values of VCL, VSL and VAP. In the spermiogram produced by CASA, the values of these parameters are indicators of velocity and reflect the hyperactivity of sperm (Peña and Linde‐Forsberg 2000). The BCF variable estimates the average frequency of path intersections by a curvilinear path and is used to identify changes in the sperm tail beating pattern (Sellés et al. 2003). Broekhuijse et al. (2012) revealed a negative relationship between BCF and birth rate. It indicates that a low BCF value during analysis suggests that the sperm is preserving energy by not fully activating its tail movements until it penetrates the zona pellucida.

Regarding sperm viability, the microencapsulation group demonstrated better results than the other group for up to 48 h of refrigeration. However, by 72 h, both tested groups were equivalent. In goat semen, a problem in cryopreservation has been the detrimental effect of seminal plasma on the viability of the spermatozoa in extenders containing milk (Leboeuf et al. 2000). In our study, we avoided this problem by using a commercial extender without milk. In addition, it would seem that microencapsulated spermatozoa are likely to be more protected against the reactive oxygen species (Gosálvez et al. 2021).

One of the most interesting findings of this study was the maintenance of sperm viability for up to 48 h in the microencapsulated group, which performed significantly better than the control group. Similar results have been reported in other species, such as cattle, humans, and swine, where microencapsulation contributed to the preservation of sperm viability, membrane integrity, and motility, in addition to reducing DNA fragmentation and prolonging sperm longevity even after storage (Kusumaningrum et al. 2015; Feyzmanesh et al. 2022; Huang et al. 2005). These findings underscore the importance of the technique, particularly because goat sperm are highly susceptible to oxidative stress during storage, which severely compromises their function (Song et al. 2025). Thus, maintaining sperm viability can positively influence post‐insemination fertility outcomes.

Various studies in different species have proven the effectiveness of artificial insemination applications. The microcapsules are filled into 0.5 mL straws for artificial insemination or cryopreservation (Perteghella et al. 2017). Studies did not observe any significant differences in the pregnancy rates in estrus synchronised females in swine (Huang et al. 2005), bovine and bubaline (Perteghella et al. 2017) after inseminating with cryopreserved‐thawed microcapsule or conventional semen straw. Studies revealed the importance of microcapsule semen over conventional straw due to its slow semen releasing ability. In our study, we did not specifically measure the timing of semen release. However, studies in different species observed in vitro slow‐releasing properties of microcapsules prepared using 1% sodium alginate (used in our study). For instance, a constant spermatozoa concentration is achieved after 24 h of incubation at 37°C, as observed by electron microscope in humans (Feyzmanesh et al. 2022). Likewise, in swine, a constant spermatozoa concentration is achieved after 24 h of incubation (Torre et al. 2002; Huang et al. 2005). This slow‐release mechanism is particularly relevant in artificial insemination protocols, as it enables a wider fertilisation window by synchronising sperm release with ovulation. Furthermore, Vishwanath et al. (1997) reported that microencapsulated bovine sperm remain viable in the female reproductive tract for up to 50 h after insemination. These findings highlight microencapsulation as an effective method for maintaining sperm quality during storage and enabling controlled release into the reproductive tract, thereby increasing the likelihood of successful fertilisation.

## 5 Conclusions

This study demonstrated the feasibility of microencapsulating goat semen. The microencapsulation treatment allowed the maintenance of sperm viability at high levels. However, further studies should investigate its effectiveness on in vivo and in vitro fertility for potential commercial applications.

## Author Contributions

F.K.P.S. was responsible for sampling and analysing the records, with oversight from M.S.C., C.H.M.R., I.M.L.A. Collaborative efforts of S.K., L.M.M. and V.J.F.F. were dedicated to critically evaluating the text and statistical results, drafting the manuscript and assessing the sources. The final version of the manuscript was reviewed and approved by all authors.

## Conflicts of Interest

The authors declare no conflicts of interest.

## Data Availability

The data that support the findings of this study are available on request from the corresponding author.
